# Immune response plays a role in *Mycoplasma pneumoniae* pneumonia

**DOI:** 10.3389/fimmu.2023.1189647

**Published:** 2023-05-26

**Authors:** Yifan Zhu, Yingying Luo, Ling Li, Xinyi Jiang, Yi Du, Jing Wang, Huilin Li, Haiyan Gu, Daiying Li, Heng Tang, Houbing Qin, Changdi Xu, Yan Liu, Deyu Zhao, Yun Guo, Feng Liu

**Affiliations:** ^1^ Department of Respiratory Medicine, Children’s Hospital of Nanjing Medical University, Nanjing, China; ^2^ Department of Respiratory Medicine, The Affiliated Wuxi Children’s Hospital of Nanjing Medical University, Wuxi, China; ^3^ Vision Medicals Center for Infectious Diseases, Guangzhou, China

**Keywords:** *Mycoplasma pneumoniae* pneumonia, microbiome, transcriptome, the immune response, *Mycoplasma pneumoniae*

## Abstract

**Introduction:**

Mycoplasma pneumoniae (MP) is a major pathogen of community-acquired pneumonia in children. However, the specific pathogenesis of the progression of Mycoplasma pneumoniae pneumonia (MPP) is unclear. We aimed to reveal the landscape of microbiota and the host immune response in MPP.

**Methods:**

This self-controlled study analyzed the microbiome and transcriptome of bronchoalveolar lavage fluid (BALF) from the severe side (SD) and opposite side (OD) of 41 children with MPP from January to December 2021 and revealed the differences of the peripheral blood neutrophil function among children with mild MPP, severe MPP, and healthy children through transcriptome sequencing.

**Results:**

The MP load or the pulmonary microbiota had no significant difference between the SD group and OD group, and the deterioration of MPP was related to the immune response, especially the intrinsic immune response.

**Discussion:**

The immune response plays a role in MPP, which may inform treatment strategies for MPP.

## 1 Introduction

*Mycoplasma pneumoniae* (MP) is a major pathogen, accounting for about 20–40% of community-acquired pneumonia (CAP) in children, with an epidemic peak occurring about every 3–7 years worldwide (1–3). Some children with *M. pneumoniae* pneumonia (MPP) develop into severe cases, presenting with various intrapulmonary and extrapulmonary complications. In recent years, the incidence of severe MPP has gradually increased in children (3). However, the specific pathogenesis underlying the progression of MPP is unclear.

Numerous studies have shown that disturbance of the microbiome is closely related to the exacerbation of many respiratory diseases, such as bronchiectasis, asthma, COVID-19, and ventilator-associated pneumonia (4–7). The pulmonary microbiome is correlated with some outcomes of MPP, such as bronchial mucus plugs and refractory MPP (8, 9). However, the relationship between the airway microbiome disruption and the progression of MPP has not been confirmed yet.

Several studies have reported increased T cell activation and high cytokine expression in the bronchoalveolar lavage fluid (BALF) from children with severe MPP (10, 11). In immunodeficient hosts, MP infection-mediated lung injury is greatly alleviated, and the probability of severe MPP decreases significantly (12). Additionally, some transcriptome studies with small sample sizes have shown that NK cells and T cells are excessively activated and proliferating during MPP (13, 14). Thus, we aimed to clarify the landscape of host response through the transcriptome analysis of BALF using a larger sample size in a self-controlled study.

The BALF of both the severe side (SD) and opposite side (OD) were collected for metagenome and transcriptome analysis. Changes in peripheral blood neutrophil function in children with MPP were revealed by transcriptome sequencing. In this study, we aimed to reveal the landscape of the microbiota and host response in MPP.

## 2 Methods

### 2.1 Patient enrollment

The inclusion criteria for children with MPP were: 1) meeting the diagnostic criteria for community-acquired pneumonia (15); 2) consistent with MP infection: MP nucleic acid test (collection of nasopharyngeal aspirates); and 3) unilateral pneumonia on chest imaging.

Children were excluded if: 1) any other pathogens were detected in the patients’ throat swabs, nasopharyngeal aspirates, sputum, BALF or blood *via* culture, viral antigen detection assays, purified protein derivative test, interferon-γ release assays, and T cell spot tests (T-SPOTs); 2) the patients had other immune deficiencies, chronic diseases, heart diseases, or were using immunosuppressive drugs; and 3) they did not agree to participate in this clinical study.

The standard of fiberoptic bronchoscopy was: after appropriate management and treatment with macrolide antibiotics, but still accompanied by long-term fever or poor absorption of chest imaging performance.

A total of 303 children with MPP admitted to the Respiratory Department of Children’s Hospital of Nanjing Medical University from January to December 2021 were prospectively enrolled. Among them, 41 children received fiberoptic bronchoscopy.

Six healthy children, six children with mild MPP, and six children with severe MPP from March to October 2018 were included to obtain purified neutrophils from peripheral blood.

### 2.2 Clinical data and sample collection

MPP in children usually presents as unilateral lesions on chest imaging, which manifest as pneumonia, pulmonary consolidation, atelectasis or pleural effusion. We defined the SD as the severe side with either of these imaging features. Fiberoptic bronchoscopy was performed under intravenous-inhalation combined anesthesia to collect BALF from the lobe with the most severe lesion on the SD and one lobe on the OD in the 41 children with MPP.

### 2.3 Purification of neutrophils from peripheral blood

Using a peripheral blood neutrophil separation kit (Tianjin Haoyang, China), neutrophils were extracted from EDTA-anticoagulated peripheral blood by density gradient centrifugation, cultured in RPMI 1640 medium containing 5% fetal bovine serum, and made into a cell suspension.

### 2.4 Nucleic acid extraction, library preparation, and sequencing

DNA was extracted from all BALF samples using a QIAamp^®^ UCP Pathogen DNA Kit (Qiagen). Human DNA was removed using Benzonase (Qiagen) and Tween20 (Sigma). Total RNA was extracted with a QIAamp^®^ Viral RNA Kit (Qiagen), and ribosomal RNA was removed by a Ribo-Zero rRNA Removal Kit (Illumina). cDNA was generated using reverse transcriptase and dNTPs (Thermo Fisher). Libraries were constructed for the DNA and cDNA samples using a Nextera XT DNA Library Prep Kit (Illumina, San Diego, CA). Library was quality-assessed by Qubit dsDNA HS Assay kit followed by a high-sensitivity DNA kit (Agilent) on an Agilent 2100 Bioanalyzer. Library pools were then loaded onto an Illumina Nextseq CN500 sequencer for 75 cycles of single-end sequencing to generate approximately 20 million reads for each library.

### 2.5 MP typing and evolutionary analysis

The fastq data, where host sequences were removed, was converted into sequences for splicing to obtain MP sequences. Then MEGA7.0 was used to conduct alignment analysis of the MP sequences and reference strain sequences by single nucleotide polymorphism (SNP) analysis and complete the drawing of the evolutionary tree.

### 2.6 Microbiome analysis

Microbial reads were obtained by mapping the data in which host sequences were removed to the ID-seq database with SNAP v1.0beta.18. The relative abundance compositions of the microorganisms in the SD group and OD group at the genus or species level were visualized. Then the relative abundance differences of the microorganisms ranked top 10 between the SD group and OD group at the genus or species level were displayed. The vegan package of R software was used to analyze the alpha and beta diversities of the microbiome.

### 2.7 Transcriptome analysis

The read count normalization and differentially expressed analyses were performed using the DESeq2 package. DEGs were determined with an adjusted P < 0.05 and absolute Log2 (fold change) > 1. The clusterProfiler package was used for Gene Ontology (GO) and Kyoto Encyclopedia of Genes and Genomes (KEGG) pathway enrichment analysis of DEGs. Benjamini-Hochberg adjusted P < 0.05 showed significant enrichment. The immune-related genes (IRGs) list was downloaded from the Immport database. According to the gene expression profile of IRGs, the Wilcoxon-Mann-Whitney rank-sum test was used to select genes that were significantly different between the two groups and visualized by pheatmap package.

To infer the composition of immune cells, the CIBERSORT algorithm with the original CIBERSORT gene signature file LM22 and 1,000 permutations were used to examine the relative proportions of 22 invasive immune cell types in each sample (16).

Based on DEGs, sequential forward selection (SFS) algorithm was used to generate the optimal gene set. We used the selected gene set to build classifiers, which were random forest model implemented using the scikit-learn package with 500 trees. Fivefold cross-validation (CV) was used to evaluate model performance. The fivefold CV was repeated 100 times, and the average result was reported.

The corrplot package of R software was used to analyze the correlation between MP load and clinical indicators or DEGs.

### 2.8 Gene set variation analysis

According to previous studies, 23 genes, including MPO, S100A8, and S100A9, and 7 genes, including Cybb, Cyba, and Ncf1, were selected to constitute the functional characteristic gene set of the neutrophil extracellular trap (NET) and NADPH oxidase (17, 18). Other functional signatures were derived from the GO database (see **Supplementary Table 1** for details). Finally, gene set variation analysis (GSVA) was performed by the GSVA package to obtain the score of each function, and the differences between groups were compared using Wilcoxon-Mann-Whitney rank-sum test and then visualized by the ggplot2 package.

### 2.9 Statistical analysis

Quantitative normal or nearly normal data are expressed as the mean ± standard deviation and analyzed by the t-test. Quantitative skewness data are presented as the median (percentile: P25, P75) and analyzed by the Wilcoxon-Mann-Whitney rank-sum test. Categorical data are expressed as the frequency and analyzed by the chi-square test.

### 2.10 Ethics and informed consent

This study was approved by the Institutional Ethics Committee of Children’s Hospital of Nanjing Medical University (Approval number: 202012089-1/201801126-1). All procedures performed in this study involving human participants were in accordance with the Declaration of Helsinki (as revised in 2013), and the informed consent of the parents or legal guardians of all enrolled children was obtained.

## 3 Results

### 3.1 Demographic and clinical characteristics

Severe or refractory MPP often requires fiberoptic bronchoscopy intervention (19). In this study, 41 children with MPP underwent fiberoptic bronchoscopy, and 262 children with MPP did not. The characteristics of these subjects are summarized in **Table 1**. Age, fever days before admission, hospitalization days, C-reactive protein (CRP), the absolute number of neutrophils, alanine aminotransferase (ALT), and lactate dehydrogenase (LDH) were significantly higher in the bronchoscopy group, and the incidence of pleural effusion and atelectasis increased significantly. In MPP, the immune response of those who required fiberoptic bronchoscopy intervention was stronger.

**Table 1:** Demographic and clinical characteristics.

		Bronchoscopy group (n=41)	Non-bronchoscopy group (n=262)	P value
Sex	Male	25 (61.0%)	143 (54.6%)	0.444
Age		6.96 ± 2.83	4.38 ± 2.80	<0.001
Fever days		7.00 (6.50,10.00)	6.00 (4.00,7.00)	<0.001
Hospitalization days		10.00 (8.00,14.00)	8.00 (7.00,9.00)	<0.001
WBC (× 10^9^/L)		9.70 ± 4.05	9.12 ± 3.64	0.352
Neutrophils (× 10^9^/L)		6.53 ± 3.16	5.15 ± 3.25	0.012
CRP (mg/L)	<8	16 (39.0%)	146 (55.7%)	0.046
	>8	17.00 (10.50,35.50)	11.00 (8.00,21.40)	
HB (g/L)		125.29 ± 11.22	126.07± 19.57	0.805
PLT (× 10^9^/L)		279.00 (224.00,351.50)	252.00 (200.00,329.00)	0.244
ALT (U/L)		18.00 (12.25,26.00)	13.00 (9.00,16.00)	<0.001
AST (U/L)		32.50 (24.50,44.00)	29.00 (23.00,35.00)	0.082
LDH (U/L)		419.00 (301.25,556.75)	323.00 (279.00,393.00)	0.002
CK-MB (U/L)		20.00 (16.25,25.75)	21.00 (18.00,26.75)	0.324
Pleural reaction	Yes	5 (12.2%)	10 (3.8%)	0.056
Pleural effusion	Yes	13 (31.7%)	11 (4.2%)	<0.001
Atelectasis	Yes	11 (26.8%)	8 (3.1%)	<0.001

Quantitative normal or nearly normal data are expressed as the mean ± standard deviation and analyzed by the t-test. Quantitative skewness data are presented as the median (percentile: P25, P75) and analyzed by the Wilcoxon-Mann-Whitney rank-sum test. Categorical data are expressed as the frequency and analyzed by the chi-square test. WBC, white blood cell; CRP, C-reactive protein; HB, hemoglobin; PLT, platelet; ALT, alanine aminotransferase; AST, aspartate aminotransferase; LDH, lactate dehydrogenase; CK-MB, creatine kinase-MB.

### 3.2 Evolutionary tree of MP genomes

To gain a deeper understanding of the molecular epidemiology characteristics of MP strains in our region, SNP analysis was used to compare the full-length sequence of MP obtained from children with MPP, whose coverage rate was more than 90%, with the whole genome sequence of MP strains publicly available in GenBank (**Supplementary Figure 1A**). Most of the MP strains sequenced in this study had high homology with MP strains sequenced in China (**Supplementary Figure 1B**).

### 3.3 MP load and pulmonary microbiome between the SD and OD had no statistically difference

**Figure 1:**
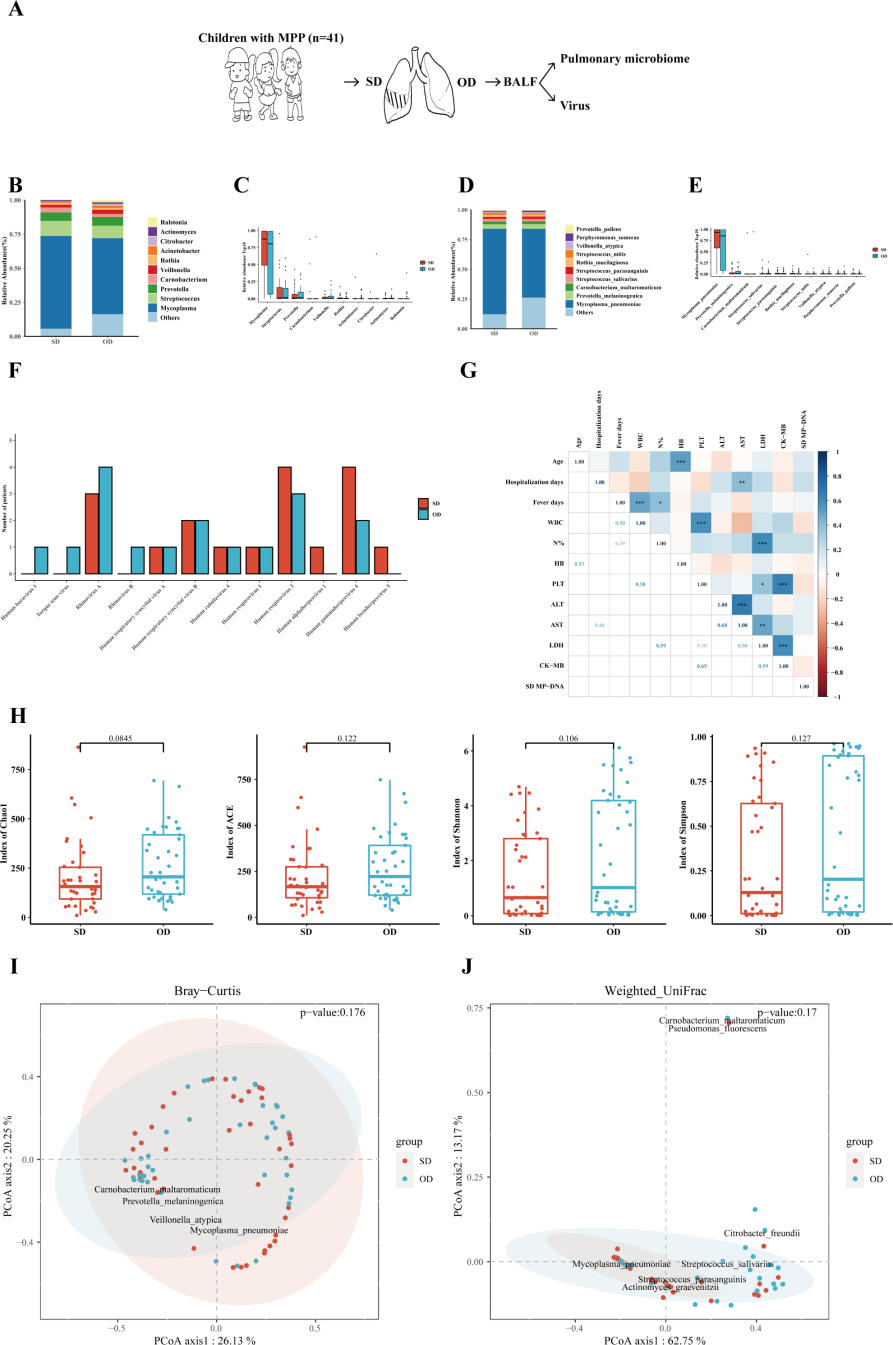
MP load and pulmonary microbiome between the SD and OD had no statistically difference. **(A)** Introduction to the specific process. **(B)** Composition of microorganisms at the genus level. The bar chart was drawn based on the relative abundance of the corresponding microorganisms at the genus level. **(C)** Comparison of the top 10 microorganisms in the relative abundance at the genus level. The relative abundance of the top 10 microorganisms at the genus level was compared by Wilcoxon-Mann-Whitney rank-sum test, and a box plot was drawn. **(D)** Composition of microorganisms at the species level. The bar chart was drawn based on the relative abundance of the corresponding microorganisms at the species level. **(E)** Comparison of the top 10 microorganisms in relative abundance at the species level. The relative abundance of the top 10 microorganisms at the species level was compared by Wilcoxon-Mann-Whitney rank-sum test, and a box plot was drawn. **(F)** Distribution of viruses in the SD and OD groups. The horizontal axis represents the name of the virus, and the vertical axis represents the number of patients. **(G)** Correlation analysis of MP reads in the SD group with clinical data. Pearson correlation coefficient was obtained by R. The color represents the size of the correlation coefficient. A *t*-test was used to test the correlation coefficient. When p < 0.05, the correlation coefficient was marked in the square of the lower left triangle, and a “*” was marked in the square of the upper right triangle; ***p < 0.001, ** 0.001 ≤ p < 0.01, and * 0.01 ≤ p < 0.05. **(H)** Comparison of the α diversity between the SD group and OD group. The ɑ-diversity index included the Chao1 index, ACE index, Shannon index, and Simpson index. The P value was obtained by Wilcoxon-Mann-Whitney rank-sum test. **(I)** Comparison of the β diversity based on Bray–Curtis between the SD group and OD group. A P value was obtained by Wilcoxon-Mann-Whitney rank-sum test. **(J)** Comparison of the β diversity based on weighted UniFrac between the SD and OD groups. A P value was obtained by Wilcoxon-Mann-Whitney rank-sum test. P values less than 0.05 were considered statistically significant.

This study evaluated the differences in the composition of pulmonary microorganisms at the genus and species levels on the SD and OD. The specific process is shown in **Figure 1A**. Amazingly, the difference of the MP load between the SD and OD was not statistically significant. At the genus level, the pulmonary microbes of MPP patients were mainly *Mycoplasma* (SD vs OD: 68.00% vs. 55.74%), followed by *Streptococcus* (SD vs. OD: 11.14% vs. 9.15%), and *Prevotella* (SD vs. OD: 6.14% vs. 6.43%) (**Figure 1B**). There was no significant difference in the relative abundance of the top 10 microorganisms between the two groups (**Figure 1C** and **Supplementary Table 2**).

At the species level, *M. pneumoniae* was dominant in the pulmonary microbes of MPP children (SD vs. OD: 71.70% vs. 57.71%) (**Figure 1D**). Similarly, the relative abundance of *M. pneumoniae* between the SD and OD groups had no statistically difference (**Figure 1E** and **Supplementary Table 3**). There was also no significant difference in the distribution of DNA viruses and RNA viruses between the SD group and OD group (**Figure 1F**). In addition, there was no significant correlation between MP reads in the SD group and most clinical indicators reflecting the severity of MPP, such as hospitalization days, percentage of neutrophils, ALT, and LDH (**Figure 1G**). However, MP reads in the OD group were significantly correlated with some clinical indicators such as ALT, AST (aspartate aminotransferase) and LDH (**Supplementary Figure 1C**).

To further quantify the differences in the microbiome, the α-diversity and β-diversity of pulmonary microbes on the SD and OD of MPP patients were compared. There were no statistically significant differences in α-diversity (such as Chao1, ACE, Shannon and Simpson index) and β-diversity (such as Bray–Curtis and weighted UniFrac distance) between the two groups (**Figures 1H–J**).

### 3.4 Neutrophil activation was more significant in the SD of MPP

**Figure 2:**
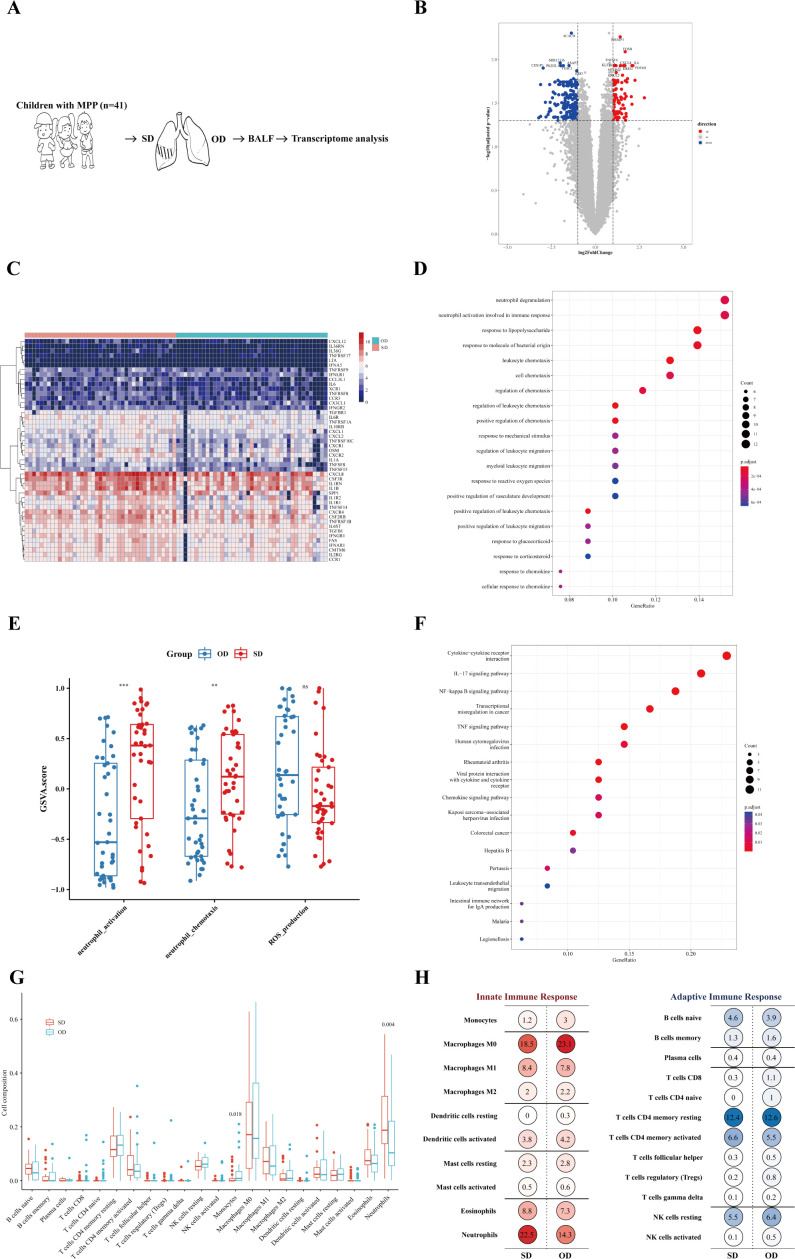
Neutrophil activation was more significant in the SD of MPP. **(A)** Introduction to the specific process. **(B)** Volcano map of differentially expressed genes. Fold change was the ratio of the average expression of genes in the SD group to those in the OD group, and an adjusted p value was obtained by the multiple hypothesis test, Benjamini-Hochberg method. The absolute value of fold change = 1 and the adjusted p value = 0.05 were used as cutoff values to determine the differentially expressed genes (DEGs). When log2 (fold change) > 1 and adjusted p value < 0.05, DEGs were considered as upregulated. The DEGs of log2 (fold change) < −1 and adjusted p value < 0.05 were considered downregulated. **(C)** SD vs. OD immune-related DEG heatmap. The heatmap is the log conversion of the DEG expression in each sample. The redder the color, the higher the DEG expression. **(D)** SD vs. OD upregulated DEG GO_BP dot plot. GO is an abbreviation for Gene Ontology. Biological process (BP) is a vital part of GO. Generatio refers to the ratio of genes enriched in this biological process to all upregulated DEGs. The size of the circle represents the number of enriched genes. Benjamini-Hochberg adjusted p value < 0.05 showed significant enrichment. **(E)** GSVA analysis. The differences in gene set scores of neutrophil activation and neutrophil chemotaxis are shown in this figure. A P value was obtained by Wilcoxon-Mann-Whitney rank-sum test. **(F)** SD vs. OD upregulated DEG KEGG dot plot. KEGG is an abbreviation of Kyoto Encyclopedia of Genes and Genomes. Generatio refers to the ratio of genes enriched in this functional pathway to all upregulated DEGs. **(G)** Proportion of pulmonary immune cells in the SD group and OD group based on transcriptome data. The CIBERSORT algorithm with the original CIBERSORT gene signature file LM22 and 1,000 permutations were used to examine the relative proportions of 22 invasive immune cell types in each sample. A P value was obtained by Wilcoxon-Mann-Whitney rank-sum test. **(H)** Average percentage of immune cells of the innate immune response and adaptive immune response in the SD group and OD group. Red represents innate immune cells, and blue represents adaptive immune cells. The darker the color, the larger the average percentage of the immune cells. *** p < 0.001, ** 0.001 ≤ p < 0.01 and ns p ≥ 0.05.

To better understand the host transcription level of MPP lesions with different severity, gene expression between the SD and OD groups of MPP patients was compared. The specific process is shown in **Figure 2A**. A total of 307 differentially expressed genes (DEGs) were identified, with 104 upregulated genes (**Figure 2B**).

The expression of 255 immune-related genes in the samples according to the ImmPortDB database was analyzed to further find a total of 47 immune-DEGs between the SD and OD groups. The expression of most chemokines and their receptors, TNF receptor superfamily, G-CSF receptor, and other genes was higher in the SD group (**Figure 2C** and **Supplementary Table 4**).

To begin with, the DEGs were significantly enriched in cell chemotaxis, especially leukocyte chemotaxis (**Supplementary Figure 2A**), in which the upregulated DEGs in the SD group were significantly enriched in neutrophil degranulation, neutrophil activation involved in immune response, and leukocyte chemotaxis (**Figure 2D**). The gene set score of neutrophil activation and neutrophil chemotaxis was significantly higher in the SD group (**Figure 2E**). In addition, KEGG enrichment analysis showed that the expression of DEGs in the SD group was significantly upregulated in cytokine-cytokine receptor interaction, IL-17 signaling pathway, NF-kappa B signaling pathway, and TNF signaling pathway (**Figure 2F**). Therefore, the aggravation of MPP may be related to a neutrophil-mediated immune response, in which IL-17, NF-kappa B, and TNF may play a role in the recruitment and activation of neutrophils, resulting in excessive immune injury.

To further explore the impact of specific immune cells in the host immune response on the worsening of MPP, CIBERSORT was adopted to find that there were significant differences in monocytes and neutrophils between the SD group and OD group, of which the SD group had a higher proportion of neutrophils, but not monocytes (**Figure 2G**). Neutrophils, macrophages, and CD4^+^ T cells dominated the lung immune cells (**Figure 2H**). This further confirmed the possible reason for the progression of severe MPP as speculated above. Neutrophil chemotaxis and activation were more significant in the SD of MPP.

### 3.5 Host transcriptional features might judge the severity of MPP

**Figure 3:**
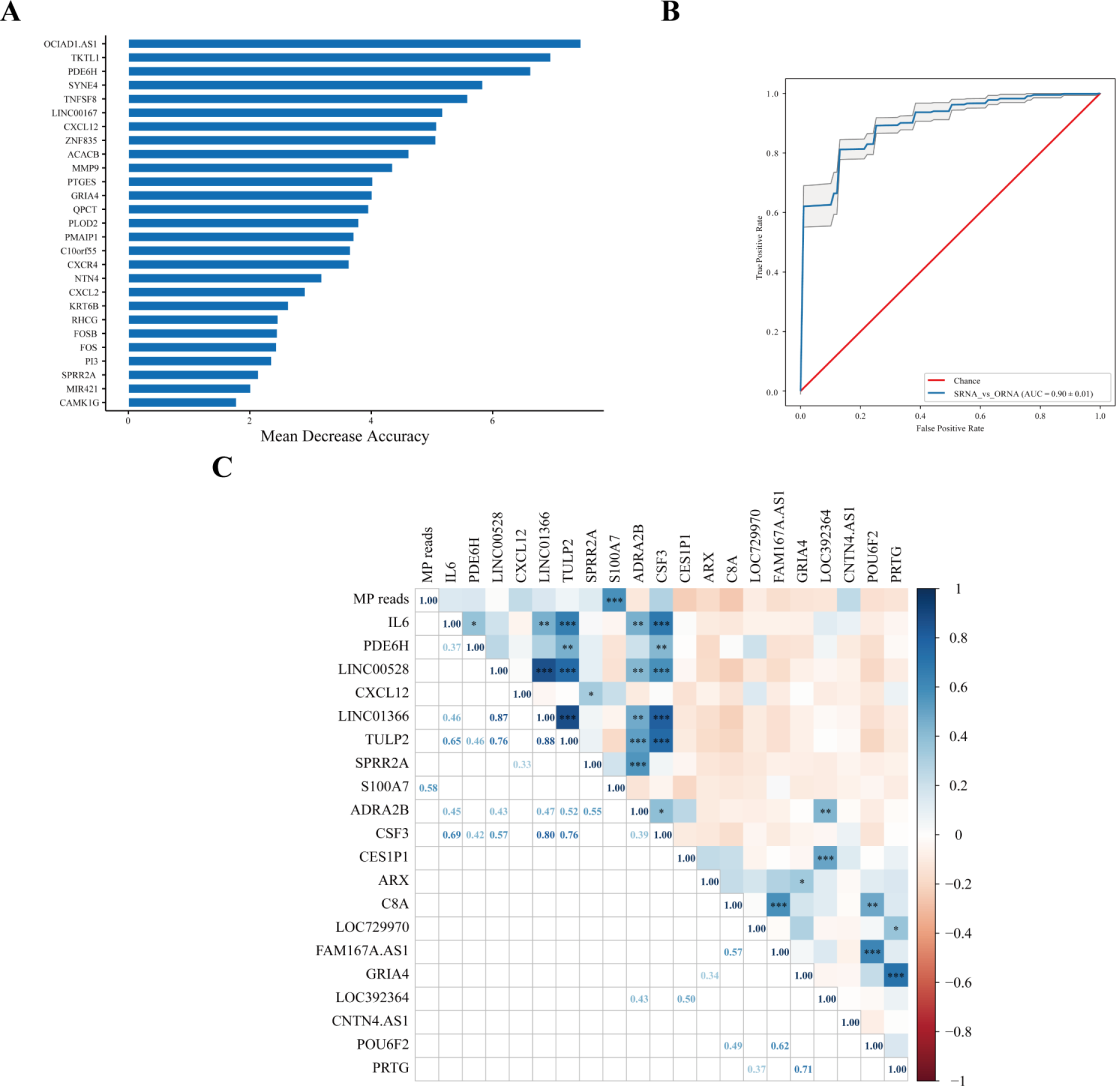
Host transcriptional features might judge the severity of MPP. **(A)** SD vs. OD feature_importance_MDA. Mean decrease accuracy (MDA) refers to the degree of reduction in the accuracy of random forest prediction. The larger the value, the greater the importance of this variable. **(B)** The ROC curve. Area under the curve (AUC) was defined as the area enclosed by the ROC curve and the coordinate axis. The closer the AUC is to 1.0, the higher the facticity of the method. **(C)** Correlation analysis of MP reads in the SD group with upregulated and downregulated top 10 DEGs. The top 10 upregulated DEGs were were obtained according to Log2FC. The 10 downregulated DEGs were obtained by the same method. Pearson correlation coefficient was obtained by R. The color represents the size of the correlation coefficient. A t-test was used to test the correlation coefficient. When p < 0.05, the correlation coefficient was marked in the square of the lower left triangle, and a “*” was marked in the square of the upper right triangle; ***p < 0.001, ** 0.001 ≤ p < 0.01, and * 0.01 ≤ p < 0.05.

A model based on host transcriptional features was established to evaluate MPP patients with more serious lesions. The greater mean decrease accuracy indicated that the DEG was more important for distinguishing the SD group from the OD group (**Figure 3A**), which was also more vital for judging the degree of aggravation of MPP. The receiver operating characteristic (ROC) analysis of the model composed of the 27 DEGs showed that the area under the curve (AUC) was 0.88 (**Figure 3B**).

There was no correlation between MP reads in the SD group and most upregulated and downregulated top 10 DEGs, except for S100A7. MP reads in the SD group were positively correlated with S100A7 (r = 0.58, p < 0.001) (**Figure 3C**).

### 3.6 Neutrophils were more significantly activated in the severe MPP

**Figure 4:**
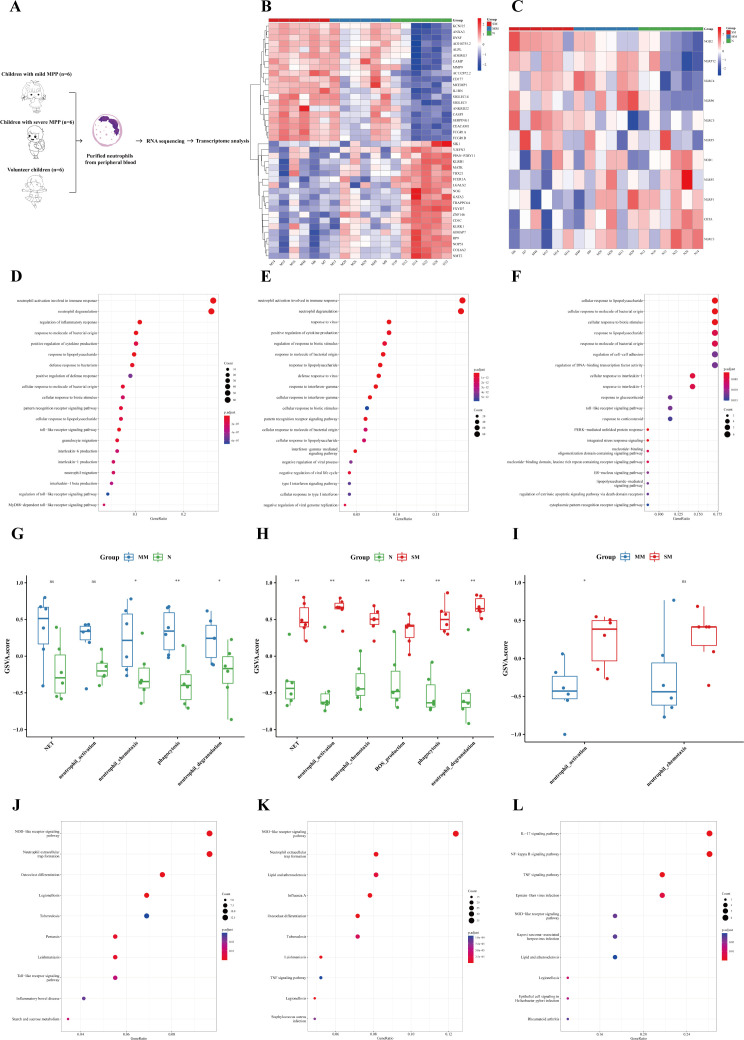
Neutrophils were more significantly activated in the severe MPP. **(A)** Introduction to the specific process. **(B)** Heatmap of differentially expressed genes between the severe MPP group, mild MPP group, and normal group. **(C)** Heatmap of differentially expressed genes of the NOD-like receiver signaling pathway between the severe MPP group, mild MPP group, and normal group. **(D)** Mild MPP group vs. Normal group upregulated DEG GO_BP dot plot. **(E)** Severe MPP group vs. Normal group upregulated DEG GO_BP dot plot. **(F)** Severe MPP group vs. Mild MPP group upregulated DEG GO_BP dot plot. **(G)** GSVA analysis between mild MPP and normal controls. The differences in gene set scores of neutrophil activation, neutrophil chemotaxis, neutrophil degranulation, phagocytosis, and NET are shown in this figure. A P value was obtained by Wilcoxon-Mann-Whitney rank-sum test. **(H)** GSVA analysis between severe MPP and normal controls The differences in gene set scores of neutrophil activation, neutrophil chemotaxis, neutrophil degranulation, phagocytosis, NET, and ROS production are shown in this figure. A P value was obtained by Wilcoxon-Mann-Whitney rank-sum test. **(I)** GSVA analysis between severe MPP and mild MPP The differences in gene set scores of neutrophil activation and neutrophil chemotaxis are shown in this figure. A P value was obtained by Wilcoxon-Mann-Whitney rank-sum test. **(J)** Mild MPP group vs. Normal group upregulated DEG KEGG dot plot. KEGG is an abbreviation of Kyoto Encyclopedia of Genes and Genomes. Generatio refers to the ratio of genes enriched in this functional pathway to all upregulated DEGs. **(K)** Severe MPP group vs. Normal group upregulated DEG KEGG dot plot. **(L)** Severe MPP group vs. mild MPP group upregulated DEG KEGG dot plot. SM, severe MPP group; MM, mild MPP group; N, Normal group. ** 0.001 ≤ p < 0.01, * 0.01 ≤ p < 0.05 and ns p ≥ 0.05.

In order to specifically explore the unique role of neutrophils in MPP, purified neutrophils were extracted from the peripheral blood of children with mild MPP, severe MPP, and healthy children for transcriptome sequencing (see **Supplementary Table 5** for details). The specific process is shown in **Figure 4A**. The DEGs among the three groups are shown in **Figure 4B**. The DEGs of neutrophils between the mild MPP group and normal group, the severe MPP group and normal group, and the severe MPP group and mild MPP group are shown in **Supplementary Figures 2C–E**, respectively. We also found that upregulated DEGs between the mild MPP group and normal group, as well as between the severe MPP group and normal group were both significantly enriched in neutrophil activation involved in the immune response and neutrophil degranulation (**Figures 4D–F**). Additionally, the gene set score of neutrophil activation was higher in the severe MPP group (**Figures 4G–I**).

KEGG enrichment analysis showed that the upregulated DEGs between the mild MPP group and normal group, as well as between the severe MPP group and normal group were both significantly enriched in NOD-like receptor signaling pathway and neutrophil extracellular trap formation (**Figures 4J, K**)). Meanwhile, the most significant upregulated gene of the NOD-like receptor signaling pathway among the three groups was NOD2 (**Figure 4C**). However, the upregulated DEGs between the severe MPP group and mild MPP group were enriched in the IL-17 signaling pathway, NF-kappa B signaling pathway, and TNF signaling pathway, which was similar to the results of the above self-controlled study of 41 children with MPP (**Figure 4L**). Thus, neutrophil-mediated immune responses may play an important role in the pathogenesis of MPP.

## 4 Discussion

This study found that the change in the MP load might not be associated with the deterioration of MPP. Furthermore, MP reads in the SD group had no significant correlation with clinical indicators reflecting the severity of MPP or the activation of immune-related genes. One study indicated that children with higher MP abundance in the BALF tend to have a longer hospital stay, higher fever peak, and higher serum C-reactive protein level (20). Another study showed that there is no statistically significant difference in the relative abundance of *Mycoplasma* between MPP children with or without bronchial mucus plugs (8).

A prospective, observational cohort study in an ICU showed that key features of the lung microbiome (bacterial burden and enrichment with gut-associated bacteria) can predict outcomes in critically ill patients (21). There was no difference in the bacterial diversity in the BALF of MPP children with or without bronchial mucus plugs (8). In an attempt to eliminate the interference of confounding factors as much as possible, our self-controlled study showed that changes in the lung microecology might not be significantly related to the deterioration of MPP. However, the comparisons between children with different severity of MPP still need to be further explored to clarify whether the MP load and pulmonary microbiota are associated with the exacerbation of MPP.

Our study further conducted transcriptome analysis to determine the factors that play a role in the worsening of MPP. We found a great difference in the immune response of BALF between the SD and the OD groups. Previous studies using T cell sorting have shown that T cell activation and cell-mediated inflammatory damage, as well as an cytokine-oriented proinflammatory environment in the respiratory tract are key components of the worsening of MPP (13, 14). Increasing evidence has also shown that neutrophil counts in the peripheral blood and BALF, as well as neutrophil infiltration in lung tissues are increased in children with severe MPP (10, 22–24). Depletion of neutrophils can reduce the severity of pulmonary lesions caused by MPP (25). Our study using BALF sequencing found that the aggravation of MPP was related to the neutrophil-mediated immune response and also indicated that the proportion of neutrophils in pulmonary immune cells increased in the SD group.

However, the role of neutrophils is a double-edged sword. Neutrophil extracellular traps (NETs) can immobilize or trap a variety of pathogens and ultimately promote pathogen killing. However, over-activated neutrophils are involved in the development of acute lung injury through respiratory bursts, degranulation, release of inflammatory mediators and NETosis (26, 27). The overproduction of NETs and particularly the NET components may cause pulmonary vascular and tissue damage (28). In chronic pulmonary inflammatory diseases such as chronic obstructive pulmonary disease (COPD), the increase of neutrophils and their release of elastase cause an increase in goblet cell numbers, mucus accumulation, and immune cell infiltration (29).

Transcriptome sequencing of neutrophils from the peripheral blood of children with MPP and healthy children further confirmed the immune results highlighting the role of neutrophils in MPP. The KEGG enrichment analysis found that upregulated DEGs between the MPP group and normal group were significantly enriched in the NOD-like receptor signaling pathway and neutrophil extracellular trap formation. NOD-like receptors are the main intracellular pattern recognition receptors, which are sensors of peptidoglycan and regulators of key inflammatory pathways, such as NF-κB and MAPK. NOD2 is significantly expressed in neutrophils and is important for the neutrophil-mediated immune response (30). Specifically, NOD2 can promote the host defense by inducing chemokines that lead to the recruitment of neutrophils and macrophages to the sites of infection and causing tissue damage (31, 32).

In summary, the deterioration of MPP was related to the immune response, which may inform treatment strategies for MPP.

## Data availability statement

The datasets presented in this study can be found in online repositories. The names of the repository/repositories and accession number(s) can be found below: HRA004226/HRA004222 (GSA).

## Ethics statement

This study was approved by the Institutional Ethics Committee of Children’s Hospital of Nanjing Medical University (Approval number: 202012089-1/201801126-1). Written informed consent to participate in this study was provided by the participants’ legal guardian/next of kin.

## Author contributions

YZ performed experiments, statistical analysis, made the figures and tables, and revised the manuscript. YLu collected clinical data, performed bioinformatics analysis, made the figures and tables, and drafted the manuscript. LL participated in the study design, collected and interpreted clinical information, and determined the clinical status for each child in the study. HG participated in the revision of the article and the supplement of the data. DL participated in the bioinformatics analysis HT, HQ and CX participated in the collection of clinical samples. XJ, HL, YD, JW and YLi participated in the collection of clinical data. DZ and YG contributed to the study design and interpretation of the data. FL designed the study, analyzed the data, and revised the manuscript. All authors contributed to the article and approved the submitted version.
