# Supplementary material for: Immune response plays a role in Mycoplasma pneumoniae pneumonia
Source: Front Immunol. 2023 May 26;14:1189647. doi: 10.3389/fimmu.2023.1189647 (PMC10250694; doi:10.3389/fimmu.2023.1189647)
Supplement: Supplementary file 1 [file DataSheet_1.pdf]

Supplementary Materials for

Immune Response Plays a Role in *Mycoplasma pneumoniae* pneumonia

Yifan Zhu *et al.*

Corresponding author. Email: zhaodeyu.98@126.com(Deyu Zhao);
guoyun@njmu.edu.cn (Yun Guo);axsliu@163.com(Feng Liu).

This PDF file includes:

Supplementary Figures 1 to 2
Supplementary Tables 1 to 5

Supplementary Figure 1

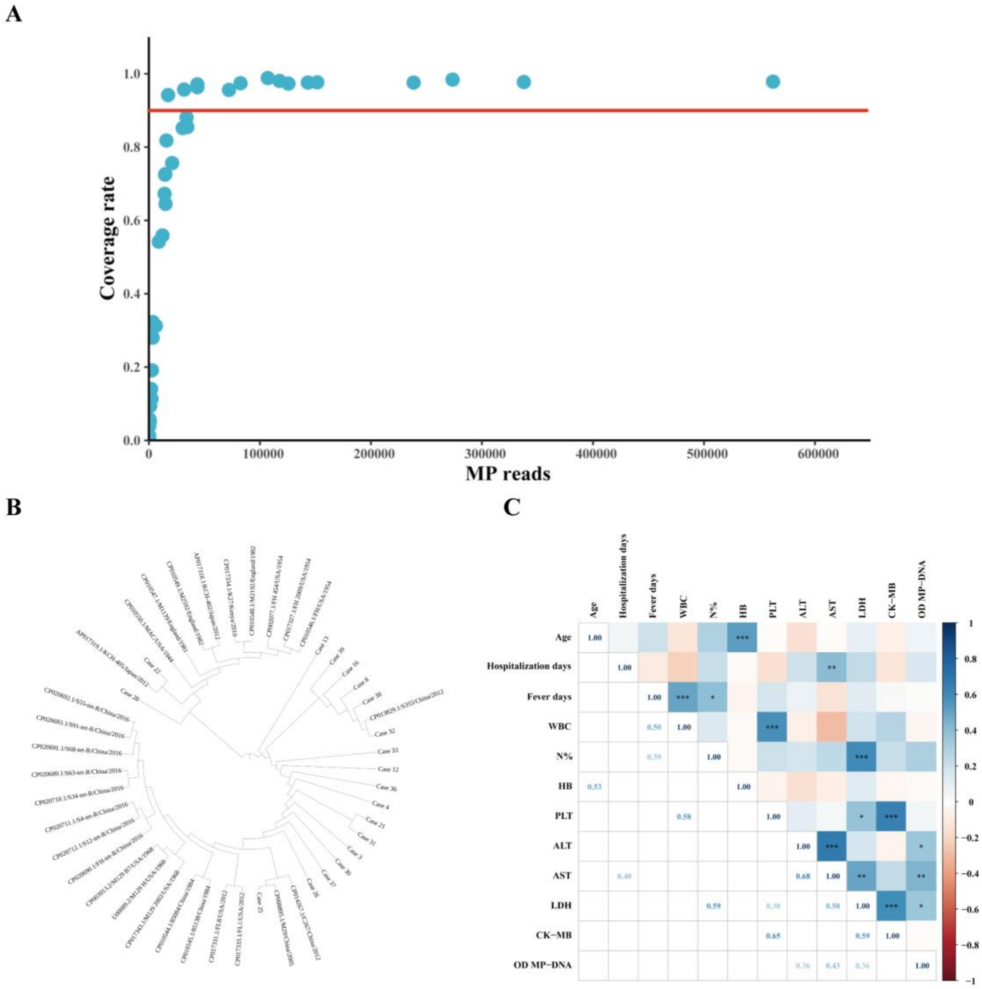

Supplementary Figure 1

(A) Scattergram of correlation analysis between MP sequence numbers and coverage rate in the SD group. The MP strains in samples whose coverage rates were greater than 0.90 were selected to draw the evolutionary tree.

(B) The evolutionary tree of MP strains. MEGA7.0 was used to conduct alignment analysis of the selected MP strain sequences and reference strain sequences and complete the drawing of the evolutionary tree. SNP analysis was performed by Neighbor-Joining Algorithm.

(C) Correlation analysis of MP reads in the OD group with clinical data. Pearson correlation coefficient was obtained by R. The color represents the size of the correlation coefficient. A *t*-test was used to test the correlation coefficient. When $p < 0.05$, the correlation coefficient was marked in the square of the lower left triangle, and a “*” was marked in the square of the upper right triangle; *** $p < 0.001$, ** $0.001 \leq p < 0.01$, and * $0.01 \leq p < 0.05$.

Supplementary Figure 2

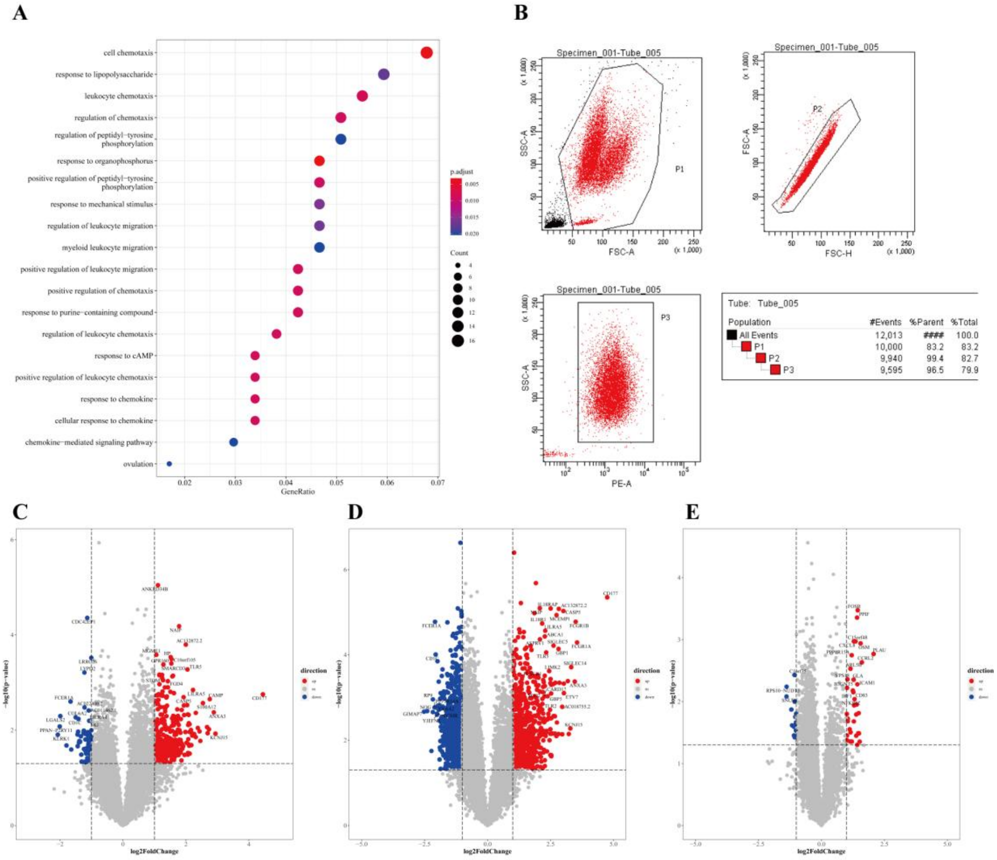

Supplementary Figure 2

(A) SD vs. OD DEG GO_BP dot plot. The dot plot was obtained from the GO enrichment analysis of all DEGs and shows the biological processes of them. The horizontal axis represents the proportion of genes associated with this function in DEGs. The longitudinal coordinate represents the relevant function. The smaller the adjusted p value, the more significant the enrichment. The size of the circle indicates the number of genes enriched to this function.

(B) Verification of the purity of the extracted neutrophils. The purity of the extracted neutrophils was determined by flow cytometry. The result was 96.5%.

(C) Volcano map of differentially expressed genes between the mild MPP group and normal group.

(D) Volcano map of differentially expressed genes between the severe MPP group and normal group.

(E) Volcano map of differentially expressed genes between the severe MPP group and mild MPP group.

Supplementary Table 1   Functional gene set.

Gene set	Gene/GO number
NETs	ACTB, ACTG1, ACTN1, ACTN4, AZU1, CAT, CTSG, DEFA3, ELANE, ENO1, KRT10, LCP1, LTF, LYZ, MNDA, MPO, MYH9, PADI4, PRTN3, S100A12, S100A8, S100A9, TKT
NADPH oxidase	Cybb, Cyba, Rac2,RAC1, Ncf2, Ncf1,Ncf4
neutrophil activation	GO:0042119
neutrophil chemotaxis	GO:0030593
neutrophil degranulation	GO:0043312
Phagocytosis	GO:0006911
ROS production	GO:1903409

The following 5 gene sets could be obtained by inputting their numbers on the Gene Ontology.

Supplementary Table 2 Comparison of the top 10 microorganisms in relative abundance between the SD group and OD group at the genus level.

Genus	P value
Mycoplasma	0.18
Streptococcus	0.59
Prevotella	0.48
Carnobacterium	0.82
Veillonella	0.45
Rothia	0.96
Acinetobacter	0.17
Citrobacter	0.05
Actinomyces	0.25
Ralstonia	0.56

The P values were obtained by a Wilcoxon-Mann-Whitney rank-sum test. A P value less than 0.05 was considered to be statistically significant.

Supplementary Table 3 Comparison of the top 10 microorganisms in relative abundance between the SD group and OD group at the species level.

Species	P value
Mycoplasma_pneumoniae	0.15
Prevotella_melaninogenica	0.49
Carnobacterium_maltaromaticum	0.67
Streptococcus_salivarius	0.76
Streptococcus_parasanguinis	0.72
Rothia_mucilaginosa	0.98
Streptococcus_mitis	0.85
Veillonella_atypica	0.71
Porphyromonas_somerae	0.90
Prevotella_pallens	0.37

The P values were obtained by a Wilcoxon-Mann-Whitney rank-sum test. A P value less than 0.05 was considered to be statistically significant.

Supplementary Table 4 Comparison of immune-related differentially expressed genes between the SD group and OD group.

Immune-related differentially expressed genes	SD vs. OD	P value
CCL3L1	Rise	0.035
CCR1	Rise	0.006
CCR3	Rise	0.022
CX3CL1	Reduce	0.039
CXCL1	Rise	0.0072
CXCL2	Rise	0.00021
CXCL8	Rise	8.8e-05
CXCL12	Rise	0.00044
CXCR1	Rise	0.008
CXCR2	Rise	0.004
CXCR4	Rise	6.5e-05
XCR1	Rise	0.0088
IL1A	Rise	0.0074
IL1B	Rise	0.011
IL1R1	Rise	0.014
IL1R2	Rise	0.0092
IL1RN	Rise	0.0071
IL2RG	Rise	0.00062
IL6	Rise	0.0031
IL6R	Rise	0.028
IL6ST	Rise	0.047
IL10RB	Rise	0.0053
IL36G	Rise	0.0051
IL36RN	Rise	0.0026
TNFRSF1A	Rise	0.033
TNFRSF1B	Rise	0.0013
TNFRSF8	Rise	0.0014
TNFRSF9	Rise	0.0073
TNFRSF10C	Rise	0.0094
TNFRSF17	Rise	0.047
TNFSF8	Rise	1.9e-05
TNFSF14	Rise	0.00074
TNFSF15	Rise	0.0062
IFNA5	Reduce	0.023
IFNAR1	Rise	0.0046
IFNGR1	Rise	0.0019
IFNGR2	Rise	0.02
IFNLR1	Reduce	0.021
TGFB1	Rise	0.0084
TGFBR1	Rise	0.015

CSF2RB	Rise	0.041
CSF3R	Rise	0.0042
CMTM6	Rise	0.0023
FAS	Rise	0.021
LTA	Rise	0.0017
OSM	Rise	0.0017
SPP1	Rise	0.00058

The P values were obtained by a Wilcoxon-Mann-Whitney rank-sum test. A P value less than 0.05 was considered to be statistically significant.

Supplementary Table 5 Demographic and clinical characteristics of children included in transcriptome sequencing test.

		Normal (n = 6)	Mild MPP (n = 6)	Severe MPP (n = 6)
Sex	Male	4 (66.6%)	2 (33.3%)	3 (50%)
Age		4.86 ± 1.88	4.40 ± 2.04	7.74 ± 3.02
WBC (× 10^9/L)		7.23 ± 0.70	9.42 ± 4.18	8.30 ± 2.86
Neutrophils (× 10^9/L)		2.12 ± 0.63	5.80 ± 3.32	5.99 ± 2.58
CRP (mg/L)	<10	6 (100%)	2 (33.3%)	3 (50%)
	≥10	0	15.00 (11.00, 23.85)	30.00, 64,98.2
HB (g/L)		123.16 ± 7.65	126.00 ± 6.32	127.17 ± 13.81
PLT (× 10^9/L)		243.17 ± 92.86	304.67 ± 200.95	274.67 ± 166.67
ALT (U/L)		12.33 ± 4.13	8.67 ± 2.73	14.67 ± 10.39
AST (U/L)		26.50 ± 2.59	23.33 ± 8.55	36.17 ± 18.68
LDH (U/L)		259.50 ± 15.49	352.33 ± 79.42	382.50 (331.00, 711.25)
CK-MB (U/L)		23.33 ± 4.18	23.00 ± 9.42	20.00 (11.50, 35.25)
Pleural reaction	Yes	/	0	2 (33.3%)
Pleural effusion	Yes	/	0	2 (33.3%)
Atelectasis	Yes	/	0	1 (16.7%)

Quantitative normal or nearly normal data are expressed as the mean ± standard deviation and analyzed by the t-test. Quantitative skewness data are presented as the median (percentile: P25, P75) and analyzed by the Wilcoxon-Mann-Whitney rank-sum test. Categorical data are expressed as the frequency and analyzed by the chi-square test. WBC: white blood cell; CRP: C-reactive protein; HB: hemoglobin; PLT: platelet; ALT: alanine aminotransferase; AST: aspartate aminotransferase; LDH: lactate dehydrogenase; CK-MB: creatine kinase-MB.
